# Techno-Economic Analysis as a Driver for Optimisation of Cellobiose Lipid Fermentation and Purification

**DOI:** 10.3389/fbioe.2022.913351

**Published:** 2022-06-17

**Authors:** Amira Oraby, Steffen Rupp, Susanne Zibek

**Affiliations:** ^1^ Fraunhofer Institute for Interfacial Engineering and Biotechnology IGB, Stuttgart, Germany; ^2^ Institute of Interfacial Process Engineering and Plasma Technology IGVP, University of Stuttgart, Stuttgart, Germany

**Keywords:** fermentation, process optimisation, techno-economic analysis (TEA), cellobiose lipids (CL), biosurfactant (BS)

## Abstract

Cellobiose lipids (CL) are glycolipids synthesized by *Ustilaginaceae* species with potential application as detergents or in cosmetics. This study identified process optimisation potential for CL fermentation based on process modelling and techno-economic analysis. Using a stoichiometric equation based on laboratory data, we calculated the maximum possible CL yield Y_P/S_ of 0.45 g_CL_·g_glucose_^−1^ at the biomass yield of 0.10 g_Biomass_·g_glucose_^−1^ with an *Ustilago maydis* strain. Due to substrate inhibition that may occur at high glucose concentrations, a fed-batch process to increase biomass and CL concentrations was considered in our model. Simulation of different process scenarios showed that the choice of aeration units with high oxygen transfer rates and adaptation of power input to oxygen uptake can significantly decrease electricity consumption. We further assessed scenarios with different fermentation media and CL purification methods, suggesting additional process optimisation potential. Here the omission of vitamins from the fermentation medium proved to be a possible mean to enhance process economy, without compromising CL productivity.

## 1 Introduction

Academic and industrial interest in microbial biosurfactants, such as cellobiose lipids (CL), has continuously increased over the past decades. This is reflected in the extensive study of their characteristics and potential applications (Banat, 1995; Banat et al., 2000; Marchant and Banat, 2012; Geys et al., 2014; Günther et al., 2017), and the recent introduction of the two biosurfactants rhamnolipids and sophorolipids to products in the cosmetic industry (Moldes et al., 2020). Within an estimated global market volume of $31B in 2016 and predicted revenue growth of 3.1% per year (Ceresana, 2017), the share of biosurfactants is expected to surpass $2.5B by 2026 (Global Market Insights, Inc., 2021). Despite this positive growth forecast, the share of microbial biosurfactants is marginal. This is mainly attributed to the high production costs of microbial biosurfactants and their low concentrations (Banat et al., 2014). At the same time, ecological concerns and the thrive towards a sustainable bioeconomy highlight the ongoing need for novel microbial biosurfactants like CL.

CLs are synthesized by microorganisms from the class *Ustilaginomycetes* using sugars as the sole carbon source (Teichmann et al., 2007; Günther et al., 2017). These glycolipids are secreted as a mixture of chemical structures that vary in their variants and mixture ratios, depending on the producing microorganisms. The backbone of CL consists of a cellobiose disaccharide with different acetylation degrees, linked to a hydroxypalmitic acid via a glycosidic bond (Lemieux, 1951; Eveleigh et al., 1964), thus showing the typical amphiphilic structure of surfactants. Further, some CL variants are reported to show antibacterial and antifungal activity towards some Gram-positive bacteria and plant pathogens (Teichmann et al., 2007; Mimee et al., 2009).

Aiming for an optimised fermentation process, several fermentation media and parameters (Oraby et al., 2020), as well as CL purification processes, were presented in the literature (Günther, 2014). However, more systematic process optimisation needs to be done to achieve an optimised CL fermentation and purification process with high titers that make CL an economically viable alternative to synthetic surfactants. In general, when it comes to cost-reduction of fermentative processes, three strategies can be followed (Banat et al., 2010):

1) Development of overproducing strains
2) Use of cheap and waste substrates
3) Development of more efficient bioprocesses, including optimisation of fermentation conditions, and downstream recovery processes

Our study followed the third approach, which focuses on process optimisation. In order to minimize the number of experimental procedures needed for process optimisation, we followed a simulation-based approach. We first modelled our fermentation and purification process using a flowsheet simulation software “basic scenario”. Then, we simulated our fermentation in a 10 m^3^ scale with several process scenarios in which different process parameters (fermentation duration, CL titer, agitation rate, aeration rate, fermentation media composition, and purification method) were varied compared to this basic scenario. Using these simulations, we conducted a techno-economic analysis (TEA) of each scenario variation to identify process bottlenecks that significantly impact the process economy. From these results, we derived the most promising process optimisation potentials that would have the most significant impact on the process economy. Others followed a similar approach for biosurfactants like sophorolipids, surfactin, rhamnolipids and other surfactants (Ashby et al., 2013; Czinkóczky and Németh, 2020; Wang et al., 2020; Ekpenyong et al., 2021; Elias et al., 2021; Moutinho et al., 2021). In comparison to their work, we present a more holistic approach that examines each process step along the whole fermentation and purification in order to identify process optimisation potential. In our approach, new scenarios were derived from the obtained results of the TEA of the basic scenario, and sensitivity analysis is only based on technical or process variations, not economic variations. This is because our aim here was to compare the effect of different variations in process parameters on the overall process economy. Using the obtained findings, we aimed at presenting recommendations that could be used for the experimental design along the process optimisation route of CL fermentation. For that purpose, relative considerations and comparisons were sufficient, whereas presenting exact economic numbers for the CL fermentation process was not within the scope of this study.

## 2 Methods

CL fermentation and purification were modeled using a flowsheet simulation software to perform a TEA of the process. Process parameters for the basic scenario were approximated based on our internal laboratory results and literature data and are referenced individually in the following. Economic evaluations were performed based on (Peters and Timmerhaus, 1991), and sensitivity analysis and scenario variations were derived based on the preliminary experimental results and obtained results of the TEA of the basic scenario.

### 2.1 Model Development and Design

Using SuperPro Designer 11.2^®^, a 10 m^3^ scaled fermenter was modelled for CL fermentation based on a process adapted after publications by our group (Günther, 2014; Oraby et al., 2020) and internal laboratory data in a 10 L scale. CL purification was done via centrifugation and ethanol extraction. All used media components are listed in Supplementary Table S1 in the supplementary.

**FIGURE 1:**
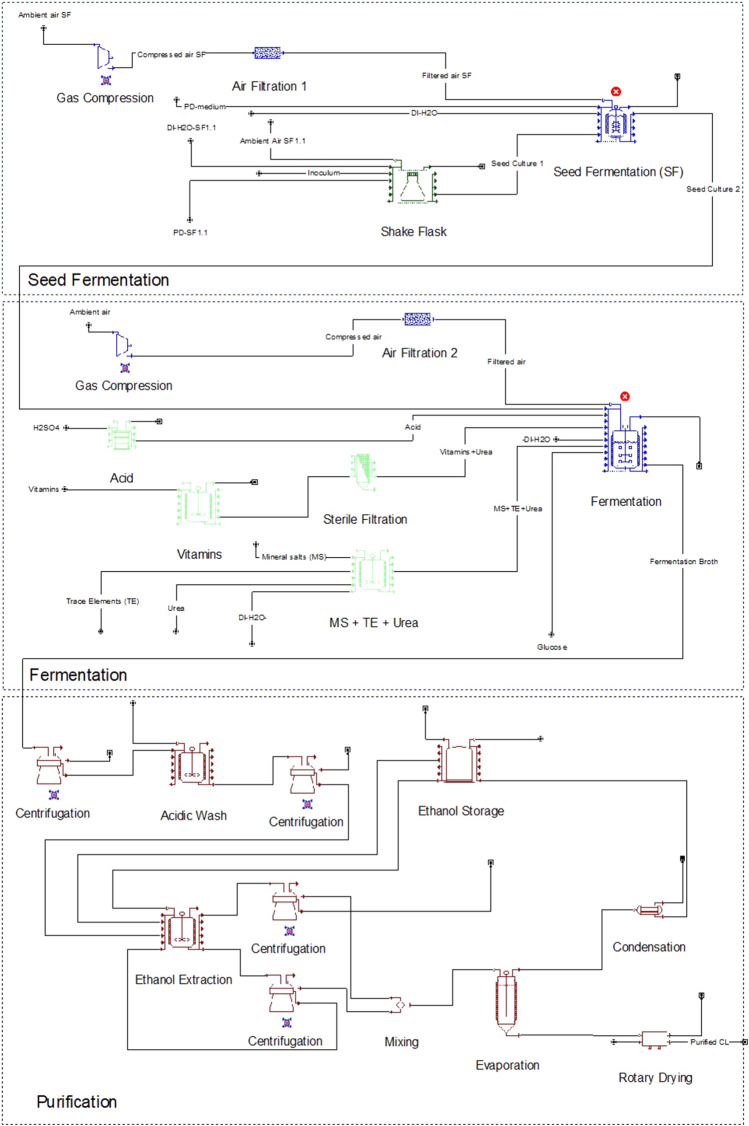
Process flowsheet for the CL fermentation, including seed fermentation, production culture, and purification units.

In the modelled fermentation, preparation of the seed culture consisted of two steps. The process flowsheet is illustrated in Figure 1. First, six 2 L shaking flasks were inoculated from potato dextrose (PD)-agar plates (Becton Dickinson, Le Pont de Claix, France) and incubated at 30°C and 120 rpm for 17 h in PD medium at pH 5.6. These were then transferred to a 300 L fermenter with PD medium to a total volume of 210 L and an optical density (OD) of 0.1. The second seed culture was agitated at 1 kW m^−3^ and aerated with 1 vvm ambient air to maintain a pO_2_ level above 20% until it reached the stationary phase after 24 h. Power input and aeration rate for the seed culture and production culture were approximated based on laboratory results (data not shown) and published results by our group for a closely related microorganism (Oraby et al., 2020). This seed culture was finally used to inoculate the 10 m^3^ fermenter containing a mineral salt medium (Supplementary Table S1) to an OD of 0.3.

0.6 g L^−1^ urea was used as nitrogen source and 100 g L^−1^ sucrose as carbon source. Sucrose is the more favourable carbon source for CL fermentation because it results in higher CL concentrations compared to glucose (Rupp et al., 2010; Oraby et al., 2020). Sucrose is hydrolysed to glucose and fructose during the first hours of fermentation and glucose is primarily consumed for CL synthesis at higher rates compared to fructose, as long as both carbon sources are available (Günther, 2014). Therefore, for approximation purposes, the hexose with highest uptake rate was used for our model and considered in the stoichiometry and yield calculations.

The production culture was agitated at 1 kW m^−3^ and aerated with 1 vvm ambient air for 14 days. 2 M H_2_SO_4_ and 2 M NaOH were used to adjust pH at 2.5. An air compressor was used to compress ambient air to 3 bars for aeration of both fermenters. All media components were heat sterilized separately, except for the vitamin fraction of the mineral salt medium. The vitamins were sterilized using a 0.2 µm filter at a 1000-fold concentration then added to the fermenter.

After fermentation, the culture broth was separated using a disk-stack centrifuge to a solid concentration of 200 g L^−1^ to obtain a wet pellet containing biomass and CL crystals. The pellet was then suspended in acidic water (pH 2.5 adjusted with H_2_SO_4_) and agitated with 1 kW m^−3^ in a stirring vessel for 30 min to wash out the remaining sugars. After the washing step, biomass and CL crystals were separated again from the suspension. In a stirring vessel, denatured ethanol was added to the pellet at a ratio of 2 g g^−1^ and agitated with 1 kW m^−3^ for 30 min. After this step, the ethanol extract containing CL was separated from the biomass using a disk-stack centrifuge. The ethanol extraction and separation steps were performed twice to ensure the extraction of all contained CL crystals from the biomass pellet. Next, both extracts were merged, and ethanol was evaporated using a thin film evaporator (at 56.6°C and 280 mbar). The crystallized CL was then dried using a rotary dryer for 24 h at 20 kg h^−1^ m^−3^.

Alternatively to ethanol extraction, a second downstream process (DSP) was also modelled. Here, the washed pellet containing biomass and CL was set to pH 12 using NaOH and incubated for 4 h at 50°C to hydrolyse CL to its water-soluble form CL-A (based on shaking flask experiments, results not shown). The biomass was then separated using a disk stack centrifuge, and the CL was precipitated using H_2_SO_4_ at a pH of 2–4. The precipitated CL-A was then separated with the disk stack centrifuge and dried for 24 h at 20 kg h^−1^ m^−3^.

### 2.2 Stoichiometry of Microbial Growth and CL Formation

For modelling and simulation purposes, the dynamic reactions that happen within the fermenter were described by mathematical equations. Hereby the reaction stoichiometry quantifies the ratio of products (CL and biomass) to the used substrates, based on the material conservation law.

For aerobic, heterotrophic microorganisms, as used here, Michael (2013) approximates the microbial growth by the following general equation:

$${\text{x}}_{1}\;{\text{C}}_{\text{a}}{\text{H}}_{\text{b}}{\text{O}}_{\text{c}}{+\text{x}}_{2}\;{\text{O}}_{2}{+\text{x}}_{3}\;{\text{NH}}_{3}{+\text{x}}_{4}\;{\text{HPO}}_{4}^{2-}{+\text{x}}_{5}\;{\text{K}}^{+}+\text{other}\;\text{educts}\to {\text{y}}_{1}\;{\text{CH}}_{\text{L}}{\text{O}}_{\text{M}}{\text{N}}_{\text{N}}{\text{P}}_{\text{O}}{\text{K}}_{\text{P}}{+\text{y}}_{2}\;{\text{CO}}_{2}{+\text{y}}_{3}{\text{H}}_{2}\text{O}+\text{other}\;\text{products}$$ (1)

C, H, N, and O are the major elements forming the general elemental composition of fungal biomass. With only 0.4–4.5%w/w taken up of phosphor, 0.1–0.5%w/w of sulfur, and even less potassium, it is plausible to neglect these elements for a first approximation for modelling purposes, as suggested by Chmiel (2006).

For CL fermentation using glucose (C_6_H_12_O_6_) as carbon source and urea (CH_4_N_2_O) as nitrogen source, Eq. 1 can thus be simplified to:

$${\text{x}}_{1}\;{\text{C}}_{6}{\text{H}}_{12}{\text{O}}_{6}{+\text{x}}_{2}\;{\text{O}}_{2}{+\text{x}}_{3}\;{\text{CH}}_{4}{\text{N}}_{2}\text{O}\to {\text{y}}_{1}\;{\text{CH}}_{1\text{.}85}{\text{O}}_{\text{0}\text{.}35}{\text{N}}_{\text{0}\text{.}03}{+\text{y}}_{2}\;{\text{CO}}_{2}{+\text{y}}_{3}\;{\text{H}}_{2}{\text{O}+\text{y}}_{4}\;{\text{C}}_{36}{\text{H}}_{64}{\text{O}}_{18}$$ (2)

whereas the elemental biomass composition is CH_1.85_O_0.35_N_0.03,_ and CL-B is represented by C_36_H_64_O_18_. Biomass compositions were determined experimentally via elemental analysis in biological triplicates and analytical duplicates (performed by the Institute of Organic Chemistry, University of Stuttgart). Setting the biomass yield Y_X/S_ to 0.2 and the product yield Y_P/S_ to 0.2 as boundary conditions and using the material conservation, the following equation A*x = b was solved to form the stoichiometry:

$$\begin{array}{c} \mathrm{Elemental}\;\mathrm{compositions}\{\begin{array}{c} \text{C}\\ \text{H}\\ \text{N}\\ \text{O} \end{array}\\ \mathrm{Boundary}\;\mathrm{conditions}\{\begin{array}{c} \text{CL}=1\\ {\text{Y}}_{\text{P}/\text{X}}\\ {\text{Y}}_{\text{P}/\text{S}} \end{array} \end{array}\overset{\begin{array}{ccccccc} \begin{array}{l} \text{Biomass}\\ \text{N}-\text{limit} \end{array} & {\text{CO}}_{2} & {\text{H}}_{2}\text{O} & \text{CL}-\text{B} & {\text{C}}_{6}{\text{H}}_{12}{\text{O}}_{6} & {\text{O}}_{2} & {\text{CH}}_{4}{\text{N}}_{2}\text{O} \end{array}}{\left(\begin{array}{ccccccc} 1 & 1 & 0 & 36 & -6 & 0 & -1\\ 1.85 & 0 & 2 & 64 & -12 & 0 & -4\\ 0.03 & 0 & 0 & 0 & 0 & 0 & -2\\ \text{0}\text{.}35 & 2 & 1 & 18 & -6 & -2 & -1\\ \text{0} & \text{0} & \text{0} & 1 & \text{0} & \text{0} & \text{0}\\ -1 & \text{0} & \text{0} & 39\text{.}5 & \text{0} & \text{0} & \text{0}\\ \text{0} & \text{0} & \text{0} & 21\text{.}8 & -1 & \text{0} & \text{0} \end{array}\right)}\cdot \left(\begin{array}{c} y_{1}\\ y_{2}\\ y_{3}\\ y_{4}\\ x_{1}\\ x_{2}\\ x_{3} \end{array}\right)=\left(\begin{array}{c} 0\\ 0\\ 0\\ 0\\ 1\\ 0\\ 0 \end{array}\right)$$ (3)

Here A is the stoichiometric matrix, x the vector of the stoichiometric factors x_i_ and y_i_, and B is the condition for the material conservation law (Chmiel, 2006).

For the seed culture, no CL is formed, and the nitrogen contained in the used complex medium is simplified by N_2_. Further, the elemental biomass composition in the seed culture is given as CH_1.83_O_0.64_N_0.13_. Here, the nitrogen content in the elemental composition is higher than the biomass during the production culture and nitrogen limitation, as discussed in detail by Klement and Buchs (2013). Therefore, the following equation was used for stoichiometric calculations of the seed culture with a Y_P/S_ of 0.2:

$$\begin{array}{c} \mathrm{Elemental}\;\mathrm{compositions}\{\begin{array}{c} \text{C}\\ \text{H}\\ \text{N}\\ \text{O} \end{array}\\ \mathrm{Boundary}\;\mathrm{conditions}\{\begin{array}{c} \text{Biomass}=1\\ {\text{Y}}_{\text{X}/\text{S}\left(\text{SC}\right)} \end{array} \end{array}\overset{\begin{array}{cccccc} \text{Biomass} & {\text{CO}}_{2} & {\text{H}}_{2}\text{O} & {\text{C}}_{6}{\text{H}}_{12}{\text{O}}_{6} & {\text{O}}_{2} & \begin{array}{c} {\text{N}}_{2}\;\text{from}\\ \text{complex}\;\text{medium} \end{array} \end{array}}{\left(\begin{array}{cccccc} 1 & 1 & 0 & -6 & 0 & 0\\ 1.83 & 0 & 2 & -12 & 0 & 0\\ 0.13 & 0 & 0 & 0 & 0 & 2\\ 0.64 & 2 & 1 & -6 & -2 & 0\\ 1 & 0 & 0 & 0 & 0 & 0\\ 0.96 & 0 & 0 & 0 & 0 & 0 \end{array}\right)}\cdot \left(\begin{array}{c} y_{1}\\ y_{2}\\ y_{3}\\ x_{1}\\ x_{2}\\ x_{3} \end{array}\right)=\left(\begin{array}{c} 0\\ 0\\ 0\\ 0\\ 1\\ 0 \end{array}\right)$$ (4)

### 2.3 Sensitivity Analysis of the Stoichiometry

In order to determine the maximum theoretically possible yields during CL fermentation, a sensitivity analysis of the reaction stoichiometry was conducted. Using Eq. 3, Y_P/S_ and Y_X/S_ were each set to a defined minimum of 0.1, while the theoretically possible maximum of the other yield was varied in steps of 0.05. The constraint here was a positive stoichiometric factor x_2_ in vector x, guaranteeing the occurrence of aerobic fermentation. If one of the two yields used for the stoichiometric matrix resulted in a negative stoichiometric factor x_2_ in vector x, that would mean that O_2_ is emitted, not consumed.

### 2.4 Economic Evaluation

For the economic evaluation, a gate-to-gate approach was followed; hence no transportation costs were calculated. Geographical aspects of the production plant were only considered for chemical and utility prices, as well as labor charges. The plant capacity was assumed as 100%, with 330 working days per year. Depreciation was calculated using the straight-line method, at an estimated depreciation period of 11 years and a salvage value of 10% (Peters et al., 2003).

Equipment purchase cost (PC) was estimated based on equipment prices from the internal SuperPro database (year of analysis 2019), except for fermenters, vessels, and filter cartridges. Here, PC was calculated using custom cost models based on price estimates from German vendors. For total capital investment (TCI) estimation, the direct fixed capital investment (FCI) was calculated based on percentages of delivered equipment cost PC (Table 1) as the sum of direct investment cost (DC), indirect investment cost (IC), and other investment costs (OIC) according to the following equations:

FCI=DC+IC+OIC (5)

TCI=DC+IC+OIC+WC+SVC (6)

**TABLE 1:** Factors applied for estimation of the direct fixed capital investment, based on the equipment purchase cost (PC), the total direct investment costs (DC), and the indirect investment costs (IC). Calculation factors are based on (Peters and Timmerhaus 1991).

	Cost Item	Factor
Direct Investment Costs (DC)	Installation	0.40 * PC
	Instrumentation	0.13 * PC
	Piping	0.31 * PC
	Electrical Installation	0.13 * PC
	Insulation	0.09*PC
	Buildings	0.29*PC
	Yard Improvement	0.15*PC
	Auxiliary Facilities	0.4*PC
Indirect Investment Costs (IC)	Engineering and supervision	0.08 * DC
	Construction	0.10 * DC
Other Investment Costs (OIC)	Contractor’s fee	0.04 * (DC + IC)
	Contingency	0.08 * (DC + IC)

The working capital (WC) investment was estimated to cover expenses for 30 operation days, while startup and validation costs (SVC) were estimated as 10 % of the fixed capital investment (Peters and Timmerhaus, 1991).

Operation costs (OC) include the costs of materials, consumables, utilities including electricity and heating/cooling agents, labor-dependent costs, costs for laboratory, quality control and quality assurance, waste treatment, facility dependent costs including maintenance, repair, and depreciation, and insurance and taxes. Prices for used chemicals and media components were obtained from bulk prices of chemical providers in Germany and are listed in Supplementary Table S1 in the supplementary. Electricity price was obtained based on the stock market price for the year 2019 in Germany, at 0.13 € kWh^−1^ (Statistische Bundesamt (Destatis), 2020). Labor costs were calculated using the built-in calculation tool of SuperPro Designer, where the total labor cost (TLC) was calculated based on the sum of labor demand per type multiplied by the labor rate per type. Here additional costs like benefits, supervision and administration were considered (Intelligen Inc., 2020). Labor demand and type were estimated based on (Peters and Timmerhaus, 1991) and calculated using average labor rates in Germany (FernUniversität Hagen, 2020). Costs for laboratory, quality control, and quality assurance were estimated as 15% of TLC. Maintenance and repair costs were estimated based on (Peters and Timmerhaus, 1991) as 6% of the TCI, while insurance accounted for 1% and local taxes for 1% of TCI, respectively.

### 2.5 Sensitivity Analysis

The unit production cost (UPC) per kg of CL was used as a scale to compare different process scenarios in our sensitivity analysis.

UPC=OC[∈]/annual CL production [kg] (7)

Based on the material and utility balances, as well as the overall process economics of the basic fermentation process (Sections 3.2 and 3.3), each cost category that is directly affected by process parameters was considered in more detail for a global hotspot analysis and scenario generation. Depreciation and facility-dependent expenditures, labour-dependent costs, and costs for laboratory and QC were not considered further because they are calculated based on equipment purchase cost and thus are not affected by simple process parameter variations. Costs for waste disposal were also not considered in the hotspot analysis.

Fermentation duration as a general parameter directly affecting process economics was varied to quantify its potential effect on CL production costs (scenarios 1–3, Table 2). Further, the CL concentration was increased to 20, 30, and 50 g L^−1^ (scenarios 4–6) at the standard yield of 0.2 g g^−1^, and the CL yield was varied to 0.3 and 0.45 g g^−1^ (scenarios 7–8) based on the maximum possible levels determined in chapter 3.1. As the primary contributor to utility demands, the aeration rate and power input were reduced from 1 to 0.1 vvm (scenario 9) and from 1 to 0.1 kW m^−3^ (scenario 10). The purification method via alkaline modification and precipitation to CL-A (scenario 11) was compared to the ethanol extraction in the basic scenario. Additionally, a fermentation process utilising a medium containing only mineral salts and trace elements without vitamins (scenario 12) was modelled. Finally, all best parameters approximated based on the current state-of-the-art values for CL fermentation were combined and simulated in scenario 13, representing a 7 days fermentation, with a power input of 0.5 kW m^−3^, an aeration rate of 0.5 vvm, using a fermentation medium without vitamins and producing 30 g L^−1^ CL at a yield of 0.2 g g^−1^.

**TABLE 2:** Overview of all varied parameters in the simulated scenarios used to estimate theoretical optimisation potentials.

Scenario	Basic	Ferm. Duration [d]				CL conc. [g/L]		CL Yield [g/g]		Aeration rate [vvm]	Power input [kW/m^3^]	CL-A	-Vita	Best
Scenario #	0	1	2	3	4	5	6	7	8	9	10	11	12	13
Duration [d]	14	10	7	5										7
Power input [kW/m^3^]	1.0										0.1			0.5
Aeration rate [vvm]	1.0									0.1				0.5
CL conc. [g/L]	10				20	30	50	15	23					30
CL yield [g/g]	0.20							0.30	0.45					0.20
CL-A	—	—	—	—	—	—	—	—	—	—	—	CL-A	—	—
-Vitamins	—	—	—	—	—	—	—	—	—	—	—	-	-Vita	-Vita

## 3 Results and Discussion

Using TEA as a driver for the choice of process optimisation approaches is an iterative process throughout the course of process development. The choice and definition of technically feasible process scenarios is a prerequisite for enabling experimental examinations of the identified optimisation approaches. Therefore, we first considered reaction stoichiometry to define the feasible range of biomass and CL yields based on their stoichiometric compositions (Section 3.1). Then material and energy balances (Section 3.2) and overall process economics (Section 3.3) of the basic scenario, which was modelled as described in Sections 2.1 and 2.2, were used to define other potential process scenarios within the predicted feasible range. In Section 3.4 the effects of variation in fermentation duration, CL concentration and yield (Section 3.4.1), aeration and agitation rates (Section 3.4.2), and the application of different purification methods (Section 3.4.3), or fermentation media (Section 3.4.4) on process economy are assessed and discussed. Based on the here obtained results, experimental optimisation approaches were suggested. Finally, a best-case fermentation scenario was simulated to assess the total reduction in CL production prices (Section 3.4.5), based on the identified optimisation potentials.

### 3.1 Determination of the Potential Maximal Y_P/S_ and Y_X/S_ Using Reaction Stoichiometry

Using Eq. 4 (Section 2.2), the stoichiometric Equation 8 in Table 3 was calculated for the seed culture, and using Eq. 3, the stoichiometric Equation 9 in Table 3 was calculated for the CL production culture. These equations were implemented in our SuperPro model to calculate material conversion during the seed culture and the CL fermentation process. Further, the sensitivity analysis for Y_P/S_ and Y_X/S_ resulted in Equations 10 and 11 (Table 3).

**TABLE 3:** Reaction stoichiometry for the seed culture (8) and potential CL fermentations with different yields Y_P/S_ and Y_X/S_ (9–11).

Y_P/S_	Y_X/S_	Reaction stoichiometry	Equation #
—	0.20	$\text{0}\text{.}96\;{\text{C}}_{6}{\text{H}}_{12}{\text{O}}_{6}+4\text{.}62\;{\text{O}}_{2}+\text{0}\text{.}065\;{\text{N}}_{2}\to 1\;{\text{C}}_{1}{\text{H}}_{1\text{.}83}{\text{O}}_{\text{0}\text{.}64}{\text{N}}_{\text{0}\text{.}13}+4\text{.}75\;{\text{CO}}_{2}+4\text{.}84\;{\text{H}}_{2}\text{O}$	(8)
0.20	0.20	$21\text{.}78\;{\text{C}}_{6}{\text{H}}_{12}{\text{O}}_{6}+37\text{.}80\;{\text{O}}_{2}+\text{0}\text{.}59\;{\text{CH}}_{4}{\text{N}}_{2}\text{O}\to 39\text{.}45\;{\text{C}}_{1}{\text{H}}_{1\text{.}85}{\text{O}}_{\text{0}\text{.}35}{\text{N}}_{\text{0}\text{.}03}+55\text{.}84\;{\text{CO}}_{2}+63\text{.}39\;{\text{H}}_{2}{\text{O}+\text{C}}_{36}{\text{H}}_{64}{\text{O}}_{18}$	(9)
0.10	0.40	$43\text{.}57\;{\text{C}}_{6}{\text{H}}_{12}{\text{O}}_{6}+18\text{.}78\;{\text{O}}_{2}+2\text{.}37\;{\text{CH}}_{4}{\text{N}}_{2}\text{O}\to 157\text{.}8\;{\text{C}}_{1}{\text{H}}_{1\text{.}85}{\text{O}}_{\text{0}\text{.}35}{\text{N}}_{\text{0}\text{.}03}+69\text{.}97\;{\text{CO}}_{2}+88\text{.}17\;{\text{H}}_{2}{\text{O}+\text{C}}_{36}{\text{H}}_{64}{\text{O}}_{18}$	(10)
0.45	0.10	$9\text{.}68\;{\text{C}}_{6}{\text{H}}_{12}{\text{O}}_{6}+4\text{.}00\;{\text{O}}_{2}+\text{0}\text{.}13\;{\text{CH}}_{4}{\text{N}}_{2}\text{O}\to 8\text{.}77\;{\text{C}}_{1}{\text{H}}_{1\text{.}85}{\text{O}}_{\text{0}\text{.}35}{\text{N}}_{\text{0}\text{.}03}+13\text{.}45\;{\text{CO}}_{2}+18\text{.}24\;{\text{H}}_{2}{\text{O}+\text{C}}_{36}{\text{H}}_{64}{\text{O}}_{18}$	(11)

The results in Table 3 show that at a maximum biomass yield of Y_X/S_ = 0.40 g_biomass_·g_glucose_^−1^, only 0.10 g CL are yielded per g glucose. On the other hand, if the biomass yield is minimised to 0.10 g_biomass_·g_glucose_^−1^, a maximum CL yield of up to Y_P/S_ = 0.45 g_CL_·g_glucose_^−1^ is possible. These theoretical considerations were used to define different scenarios analysed in Section 3.4.

At a given glucose concentration of 50 g L^−1^ in the culture medium, as set for the basic CL fermentation scenario, a product yield of 0.45 g_CL_·g_glucose_^−1^ would mean that in batch fermentation, no more than 22.5 g L^−1^ CL can be produced. However, to improve the process economy, higher CL concentrations are necessary (see Section 3.4). At the same time, at a maximum yield of 0.45 g_CL_·g_glucose_^−1^, initial substrate concentration needs to increase significantly to achieve higher concentrations. An increase in initial hexose concentrations is however limited since osmotic stress, and byproduct synthesis increase with higher substrate concentrations in the medium and may result in a decrease in productivity and product yield (Krull et al., 2020). In a previous work, we showed that increasing the glucose concentration from 50 to 100 g L^−1^ resulted in a decrease in CL concentration and yield, as well as an increase in byproduct formation with *Sporisorium scitamineum*, a close relative to the strain used in this work (Oraby et al., 2020). Therefore, applying a fed-batch process, where additional carbon source, such as sucrose, is supplied during the fermentation, while the overall level of hexoses is held below 50 g L^−1^ at all times, may be the more suitable solution to obtain high concentrations without decreasing product yields (compare results in Section 3.4.1).

Comparing the here obtained theoretically maximum possible concentration with literature values shows that there is still optimisation potential for increasing the CL concentration. A maximum of 23 g L^−1^ CL was reported in literature by Roxburgh et al. (1954) for batch fermentation, with an initial glucose concentration of 100 g L^−1^, resulting in a Y_P/S_ of only 0.23 g_CL_·g_glucose_^−1^. Günther showed in his work that this maximum concentration could be increased to 33 g L^−1^, using sucrose as the carbon source in a fed-batch process, thus illustrating the potential increase in CL concentration through substrate feeding (Günther, 2014).

### 3.2 Process Model and Overall Material and Utility Balances of the Basic Scenario

Implementing Eqs 5 and 6 from Section 2.2 to describe the fermentation stoichiometry for both the seed and production cultures, the CL basic fermentation process scenario was modelled in SuperPro Designer according to the procedure described in chapter 2.1. All equipment used for the CL model is listed in Supplementary Table S2 in the supplementary section.

Using Eq. 6 and an initial glucose concentration of 50 g L^−1^, a CL concentration of 10 g L^−1^ is calculated for the basic scenario, resulting in 70 kg of CL per batch, amounting to 1,473 kg CL per year in the 10 m^3^ fermenter. Equally, 71 kg biomass per batch and 1,488 kg per year are produced. When product loss during DSP is considered, a final amount of 57 kg CL per batch and 1,201 kg CL per year are yielded. These target product amounts were used for economic calculations. 222 kg of carbon dioxide is emitted per batch due to microbial metabolism, while 109 kg of oxygen is consumed. Besides glucose (350 kg/batch), water, and sodium hydroxide, which are mainly used for CIP procedures, the other large material stream is ethanol, used for CL extraction. Here, an average of 1,534 kg ethanol is used per fermentation process, however only 248 kg of which are lost during DSP. The losses in ethanol occur mainly during the drying step of CL. The remaining 1,286 kg are regenerated during the evaporation step of the CL extract and can be used for CL extraction in proceeding batches.

Besides the material’s demand, the consumption of utilities, including electricity, steam, cooling, and chilled water, was calculated for all process steps. In our model, electricity costs were the major contributor to utility costs. More than 99% of electricity was consumed by the production culture fermentation section, of which 65% were used for the compressor that provides compressed air to the fermenter for aeration, and 15% were utilized for fermenter agitation. In comparison, only 0.16% of electricity demand was utilized in the DSP section. Chilled water (5°C supply temperature and 10°C return temperature, in an assumed closed cycle loop) amounted to the second-largest cost contributor to utility costs, 97% of which were consumed by processes related to the production culture fermentation. Approximately half of the chilled water was used to temper the air compressor. The second half was used for general temperature control during fermentation and after sterilisation. Cooling water (25°C supply temperature and 30°C return temperature) was exclusively used to cool down the separators used for DSP. 70% of the consumed steam was used for fermenter heating and sterilisation processes and 28% during DSP for evaporation and drying. More than 98% of utility cost shares are attributed to the fermentation process and media preparation, with less than 2% for DSP. These results contradict with literature stating an overall major cost-share for DSP processes up to 60% (Banat et al., 2014). However, in our case, the comparably small share of DSP processes in utility costs may be due to the relatively long fermentation duration and the herewith associated high utility demand for the fermentation section. Furthermore, the purification process required to obtain CL with ethanol extraction does not need costly equipment or materials. A reduction in fermentation duration would nevertheless result in a relative increase in the cost-share of DSP.

### 3.3 Overall Process Economics

TCI and OC were calculated according to chapter 2.4 for our basic CL fermentation scenario in a 10 m^3^ fermenter, resulting in a TCI of € 5.85 million and OC of € 1.19 million per year. These numbers are based on a pilot-scale plant and thus do not reflect realistic overall economic costs of an industrial plant; however, they are used as a baseline to compare different potential fermentation scenarios. Therefore, all further considerations will be evaluated relatively and not as absolute numbers. The pilot-scale simulation was chosen as a first scale-up step for better approximation. For further techno-economic analyses, more detailed process data needs to be obtained from pilot-scale fermentations to increase model accuracy for larger scales.

The major share of annual operation costs for the basic CL fermentation scenario was caused by the purchased equipment, expressed in depreciation and other facility-dependent costs (>70%). Labour-dependent costs were the third major contributor, accounting for 17.42% of OC. This value is within the range of traditional chemical plants and biorefineries with single output systems (Peters and Timmerhaus, 1991; Jorissen et al., 2020). Utility costs accounted for 5.47% of the OC for CL production and are considered within range compared to literature values for biorefineries, covering a wide range from 6% (e.g., Gómez-Ríos et al., 2017) up to 41% (e.g., Mussatto et al., 2013). Laboratory costs amounted to 2.61% of the OC and raw materials to 2.27%. The laboratory costs are within range, compared to chemical plants, as indicated by Peters and Timmerhaus, whereas raw material costs vary from the typical range of 10–60% of the total product cost (2003). This comparably small share in this study may be explained by the high facility-dependent costs for the modelled fermentation due to the relatively small plant scale with a fermenter scale of only 10 m^3^. At a higher production and larger plant scale, raw material cost shares usually increase, with the absolute minimal production cost majorly caused by the raw material costs of the main substrates (Banat and Thavasi, 2018). An economic analysis for sophorolipid fermentation at a scale of 19,832 m^3^ estimates up to 89% of the operation costs for raw materials (Ashby et al., 2013). The smallest shares of operation costs in the CL model were caused by consumables and waste disposal at 0.08% and 0.08%, respectively. However, it must be noted that only the disposal cost for the alkaline water used for reactor cleaning was considered. For consumables in biorefineries in general, values between 1% up to 14% of operation costs are usually spent (Jorissen et al., 2020).

### 3.4 Sensitivity Analysis (Scenarios, Derived From 3.2)

Based on the analysed overall process economics of the basic scenario, different scenario variations were simulated in order to assess the optimisation potential for CL fermentation. The effect of variation of the different parameters listed in Table 2 on the UPC of CL is presented and discussed in relation to potential process optimisation approaches in the following.

#### 3.4.1 Variation of Fermentation Duration, Concentration, and Yield

The fermentation duration of the production culture was set to 14 days in our basic scenario, based on our internal experimental data. The glucose fraction obtained from sucrose hydrolysis is usually consumed after approx. 5 days of fermentation at a starting level of 100 gL^−1^ sucrose. After that, fructose is metabolized at lower rates compared to glucose and is completely consumed after up to 14 days and with lower CL production rates (own experimental data, not shown). This shows that higher CL concentrations are achieved after longer fermentation durations at lower productivity rates. To design an economic process, it is therefore essential to compare the effect of a high concentration to the effect of shorter duration on process economy since a combination of both is not achievable.

**FIGURE 2:**
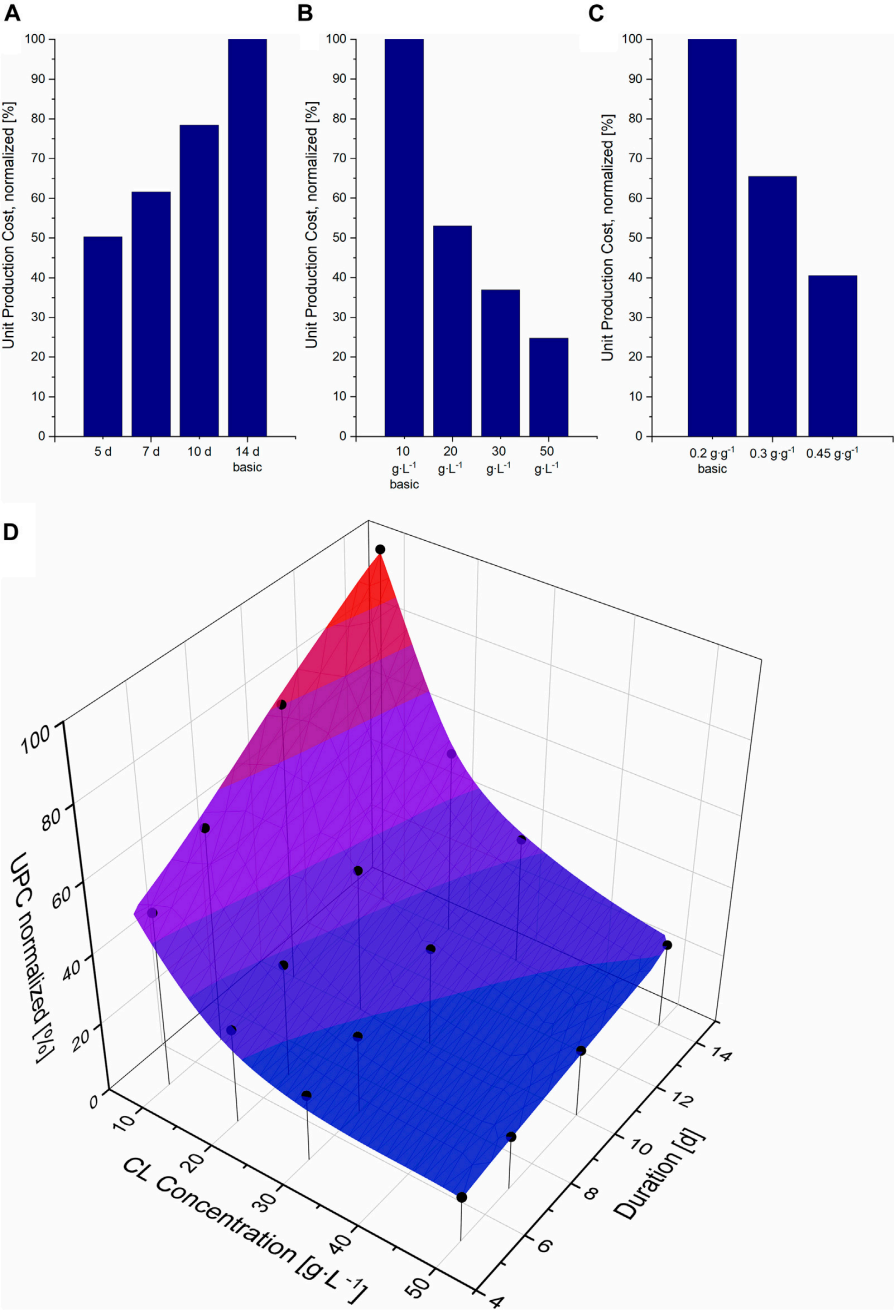
Unit production cost (UPC) per kg CL, normalized to the UPC for the basic scenario (14 days fermentation, 10 g L^−1^ CL at a yield of 0.2 g g^−1^), compared to different fermentation durations **(A)**, different CL concentrations **(B)** and yields **(C)**. **(D)** Surface cubic spline interpolation and simulated data points for the UPC at different fermentation durations and CL concentrations. The simulated data points are illustrated as black circles.

Reducing the fermentation duration from 14 to 5 days in our model allowed more batches per year. This increased the plant’s productivity while the investment costs remained the same and only the operation costs increased, thus resulting in a reduction in UPC (Figure 2A). Assuming the same CL concentration of 10 g L^−1^, an increase in fermentation duration results in a linear increase in UPC. To assess the effect of higher concentrations, three higher concentrations were further calculated for the standard fermentation duration of 14 days (Figure 2B). Here, a fed-batch process had to be simulated to obtain the higher concentrations at the same yield of 0.2 and with 50 g L^−1^ glucose in the initial medium. Depending on the needed feeding rate, the initial volume had to be decreased for these scenarios. An increase of CL concentration to only 20 g L^−1^ resulted in a reduction of 47% of UPC, almost as much as the reduction of fermentation duration to 5 days. A larger decrease in UPC could theoretically also be achieved, in a batch process, if the product yield was increased to 0.45 g g^−1^, also resulting in a CL concentration of 22.5 g L^−1^ (Figure 2C). The decrease in UPC is higher when increasing the substrate yield instead of feeding more substrate, mainly because of the smaller vessel sizes for DSP and thus the lower PC. With a higher CL yield at the expense of biomass yield, the pellet size that needs to be purified is smaller than the pellet in a fed-batch process where the biomass yield remains the same. Thus vessels needed for DSP are smaller, the needed solvent amount for extraction and the amount of CL entrapped in the biomass pellet after extraction are less. The contribution of the additional substrate cost, in that case, has a less significant effect.

This result contradicts common literature data, where usually the substrate costs take a significant share of operation costs (Ashby et al., 2013; Cheng et al., 2017; Klein et al., 2017; Wang et al., 2020). However, the plant capacity modelled in this case study can easily explain this apparent contradiction. At a fermenter size of only 10 m^3^, the revenues cannot be in a positive relation to the investment costs, especially not at such low concentrations of CL. Regular production plants would have much larger capacities, and the scaling effect would decrease the share of depreciation and PC-related expenses in favour of an increase in the share of running costs like substrate consumption. Therefore, although an increase in yield is limited to a maximum of 0.45 g g^−1^, for future perspective, it would be advisable to aim at reaching this maximum CL yield as well. This could be achieved either by genetic modification, for instance, or by fermentation media adaptation (Banat et al., 2010).

When comparing the effect of a shorter fermentation duration to the effect of higher CL concentrations on UPC, favouring one option over the other is not trivial, and combining both aims cannot be achieved in practice. As previously shown in Section 3.1, CL concentrations higher than 22.5 g L^−1^ can only be achieved through a fed-batch process. On the other hand, substrate consumption rates show that the initial glucose (at a starting level of sucrose of approx. 100 g L^−1^) is completely consumed after approx. 5 days, and the fructose even later. This means that a fed-batch process cannot be implemented for such short fermentation durations and would be only applicable for longer fermentation durations. Therefore, to identify the most promising parameter combination for an economic fermentation process that can be realised, 16 data points were simulated and a cubic spline interpolation was calculated with Origin (Figure 2D).

Comparing both the effect of time variation and concentration on UPC, it is apparent that an increase in CL titer results in a larger reduction in UPC compared to a decrease in fermentation duration. This is especially the case at lower CL concentrations. The higher the CL titer, the less the decrease in UPC. This is due to the additional equipment PC needed for larger DSP units like solvent vessels and extraction tanks. On the other hand, the effect of fermentation duration is linear, as no additional costs are needed for shorter fermentation durations, but more batches per year can be operated, and thus the plant’s capacity can be increased. In other words: approximately the same reduction in UPC can be achieved by reducing the fermentation duration from 14 to 5 days with a titer of 10 g L^−1^ or by doubling the concentration from 10 to 20 g L^−1^ at the same fermentation duration of 14 days.

#### 3.4.2 Variation of Aeration Rate and Power Input for Agitation

**FIGURE 3:**
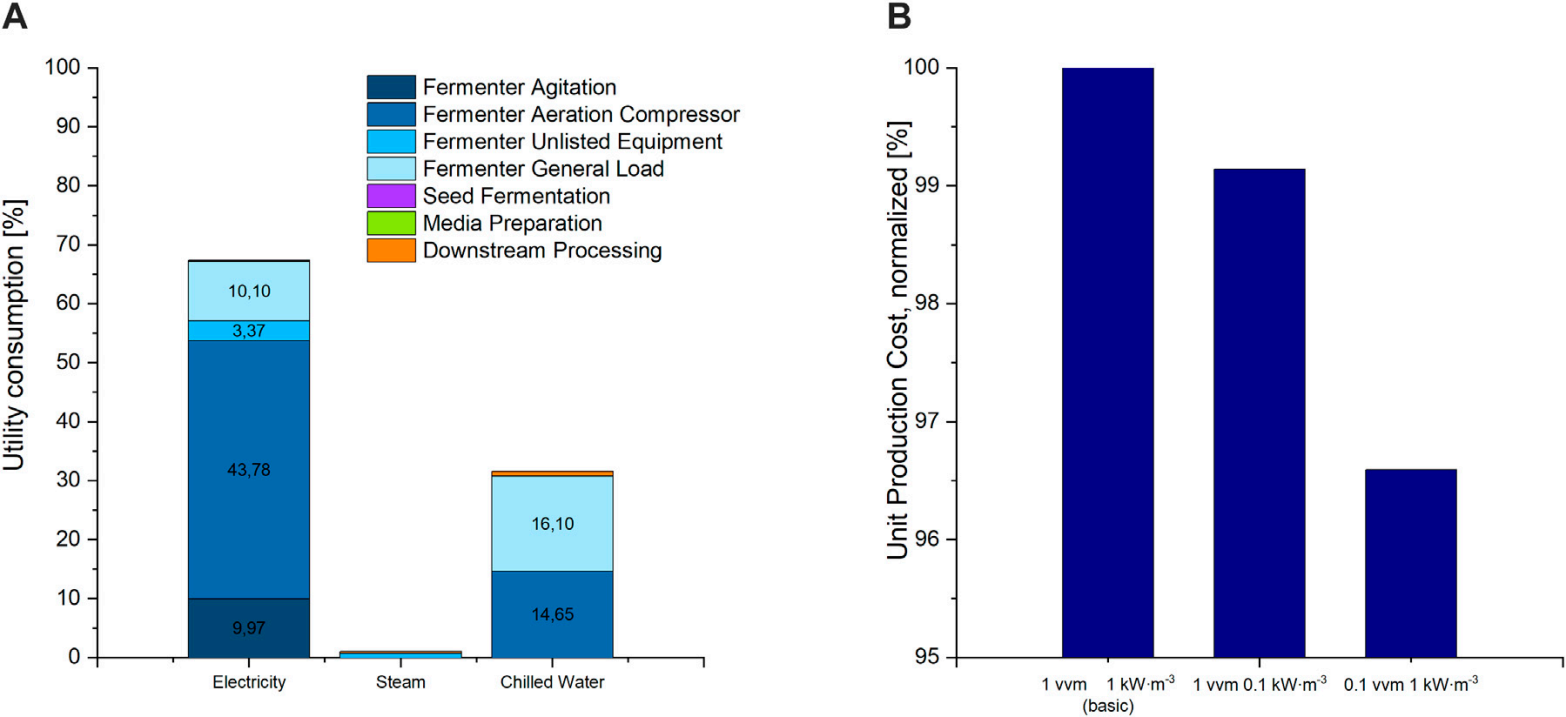
**(A)** Distribution of utility costs for electricity, steam and chilled water used for cooling amongst all fermentation and purification units, as percent of overall utility consumption costs. Shares below 3% are not labelled in the diagram. **(B)** UPC of the basic scenario at a power input of 1 kW m^3^ for agitation and an aeration rate of 1 vvm compressed air, compared to scenario 11 with 0.1 kW m^3^ and scenario 10 with 0.1 vvm.

Utility costs, making up the highest share in OC of the process-parameter dependent cost categories, are mainly caused by electricity consumption during fermentation (Figure 3). In a TEA of a rhamnolipid production process, Moutinho et al. also showed that electricity demand makes up a major share of variable costs (2021). In our model the major share of electricity cost is taken up by the compressor used for fermenter aeration, followed by the consumption of the stirrer engine for fermenter agitation. In aerobic fermentation processes, both the agitator and stirrer are used to supply the medium with the oxygen needed by the microorganisms. Gas bubbles are introduced to the liquid via a gas sparger, the agitator. Then they are dispersed into smaller bubble sizes to provide a larger surface for oxygen transfer from the gas, into the liquid phase and to the microorganisms (Doran, 2013). The stirrer is further used for medium homogenization. The minimum power input to the stirrer and the aeration rate are limited by the oxygen demand of the microorganisms.

Based on the high contribution of both the stirrer and aeration compressor to utility consumption, a reduction in both or either should result in a reduction in operation costs. Reducing the power input from the initial 1 to 0.1 kW m^3^ and the agitation rate from 1 to 0.1 vvm however resulted only in a slight decrease in UPC of less than 1% and 4%, respectively (Figure 3). This can be explained by the overall low share of utility costs to the overall OC of less than 5.5%. Nonetheless, considering environmental aspects, reducing electricity consumption during fermentation should be aspired. Emissions to the environment due to electricity generation seem to have a high share in environmental impact in biosurfactant fermentations (Aru and Ne, 2018; Kopsahelis et al., 2018). Therefore, for experimental optimisation, both power input and aeration rate should be adjusted to only cover the microorganism’s oxygen demand without providing an excess in oxygen supply. Fermentation studies show a non-constant oxygen demand during CL fermentation (Oraby et al., 2020). Thus, a stepwise gas supply cascade can be applied, with high oxygen supply during exponential growth and reduced supply afterward and in the lag phase. This would result in an overall reduction of utility demand for oxygen supply.

To design such a gas supply cascade, maximal oxygen demand by the microorganisms (OUR_max_) needs to be determined experimentally in a first step. Complementary to adapting the oxygen supply to the oxygen demand, the choice of a sparger unit can further result in a decrease in power consumption due to agitation and aeration. Using a ceramic sparger unit with small pore sizes, instead of traditional aeration rings, proved to be a viable option to reduce the power input in a 10 L fermenter without compromising the oxygen supply to the microorganisms (exemplary kLa values are shown in Supplementary Table S3). This shows that decreasing the electricity demand for agitation and aeration can easily be achieved by a better understanding of the fermentation process and adequate design of air supply cascades.

#### 3.4.3 Comparison of Different Purification Processes

**FIGURE 4:**
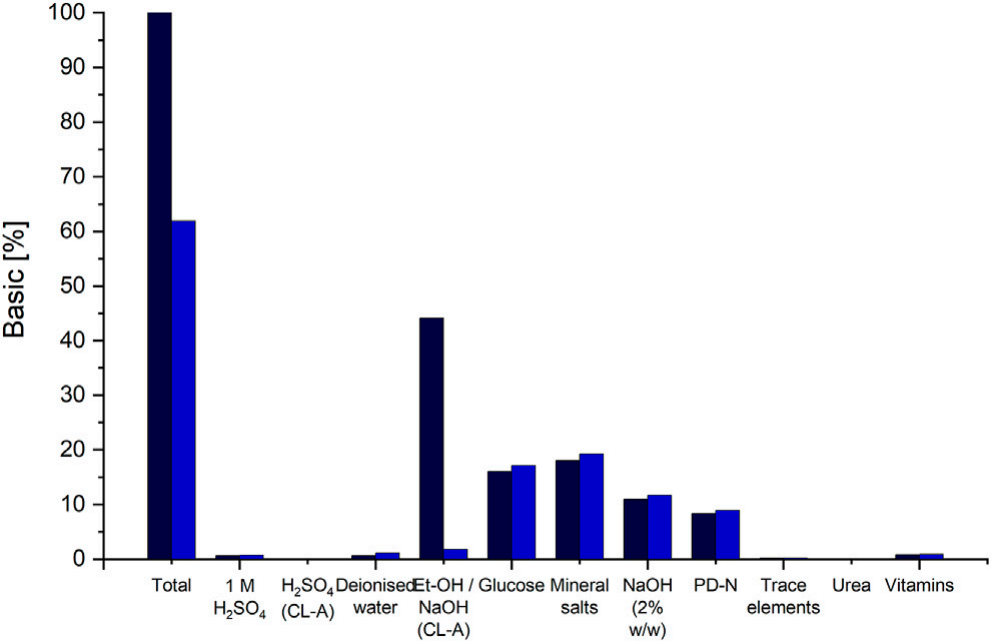
Distribution of raw material cost-shares on the different chemicals and substrates used for fermentation and DSP. The cost shares of the basic scenario are indicated via the black columns and the cost shares for scenario 12, applying the DSP method using alkaline hydrolysis and precipitation to CL-A, are indicated in blue. The column “Et-OH/NaOH (CL-A)" indicates the cost shares of ethanol, for the basic DSP method and the cost shares for NaOH in scenario 12.

The commonly applied purification method for CL is solvent extraction (Hammami et al., 2008; Morita et al., 2011; Günther, 2014). In our basic scenario, we modelled a DSP process using ethanol to extract CL. Here, the ethanol is added to the pellet in a ratio of 2 g_EtOH_·g_Pellet_^−1^. The pellet contains after separation approx. 80% water and only about 10% CL and 10% biomass. This results in massive consumption of ethanol per g CL during extraction. Consequently, due to the large volumes, EtOH amounts for the largest share (>40%) of material costs (Figure 4). A reduction of the amount of used ethanol is not feasible because of the high water content in the pellet. Internal solubility examinations using a saturation shake-flask method adapted after (Baka et al., 2008) showed a decrease in CL solubility in ethanol at water contents above 40% (results not shown). Therefore, to ensure maximal CL solubility at room temperature, a minimal amount of 2 g_EtOH_·g_Pellet_^−1^ has to be used for extraction.

If an ethanol-free purification method, namely CL hydrolysis and purification via pH shift, is applied, material costs can be reduced by approx. 38% (Figure 4). The amount for all media components remains the same, except for NaOH and H_2_SO_4,_ used instead of ethanol. These however result in overall lower material costs compared to the costs of ethanol. Together with an associated reduction in equipment PC, since no evaporation and condensation would be needed, a total reduction in UPC by approx. 18% can be achieved by using the DSP method via pH shift. However, it has to be noted here that this would result in a different CL structure (CL-A), which needs to be considered when choosing the adequate purification method. Another means to reduce raw material costs is recirculating the evaporated ethanol after its condensation and reusing it for CL extraction.

#### 3.4.4 Comparison of Different Fermentation Media Compositions

The choice of fermentation media components can affect both biomass growth and CL formation, as well as process economics. One approach for fermentation media optimisation is following experimental design techniques (Banat et al., 2010). Although highly beneficial, such examinations require many experiments and are very time-consuming. At the same time, economic considerations are usually neglected in such approaches. Considering economic factors of individual media constituents may limit the number of necessary experiments, especially if the omission of unnecessary media components is aspired by the experimental design. Here, the omission of more costly components first may reduce the number of needed experiments that have the most significant effect on both process economy and growth and CL formation.

The distribution of raw material cost shares shows that fermentation media components have comparably low shares (Figure 4), with both glucose and mineral salts contributing with approx. 16% and 18% each. Substituting glucose with other carbon sources originating from waste materials such as agro-industrial side streams may be a feasible option for cost reduction (Banat et al., 2010). However, mineral salts are necessary for both growth and CL formation and can therefore not be omitted (Oraby et al., 2020). The complex PD-N medium is needed for seed fermentation and cannot be omitted. All other media components used for the production culture have a rather small contribution to raw material costs. However, the preparation of individual media component groups may require additional equipment or consumables.

**FIGURE 5:**
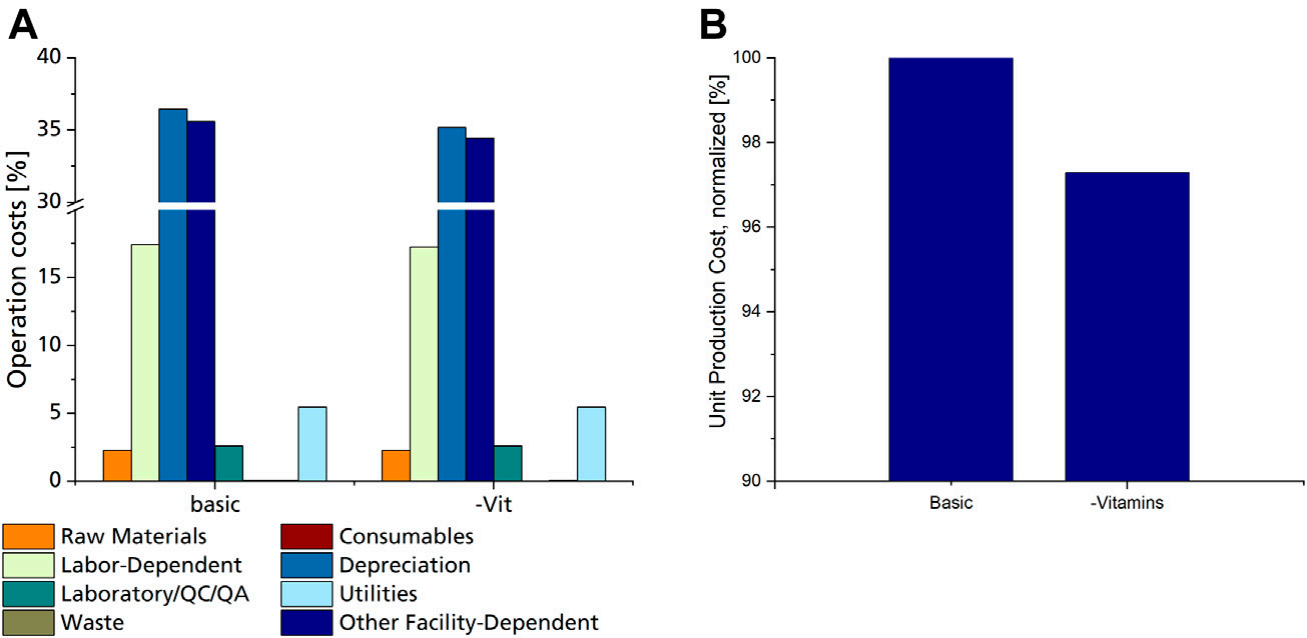
**(A)** Distribution of operation costs for the basic scenario and scenario #13 utilizing a fermentation medium without vitamins, as % of overall operation costs. **(B)** Normalized UPC of the basic scenario with mineral salt medium, including vitamins, compared to scenario 13 utilizing the same mineral salt medium without vitamins.

Consumable costs account for only 0.08% of OC, of which filter cartridges used for sterile filtration of media components amounted to 97%, while the rest was calculated for shaking flasks used for seed culture cultivation. Looking closer at the used media components, it is evident that only heat-labile vitamins need to be sterile filtrated, while the rest of the components can be heat sterilised. Heat sterilisation does not require any additional consumable materials and can be done in regular tanks. This means that although vitamins have a negligible share in raw material costs, their omission from the fermentation medium would result in a cost reduction since filters and filter cartridges for sterile filtration will not be needed. A decrease of up to 2.7% in unit production cost can be achieved by omitting vitamins from the fermentation medium (Figure 5B). However, this decrease is only valid if CL production is not affected by the lack of vitamins in the fermentation medium. This proved to be the case when comparing CL productivity with *Sporisorium scitamineum* on the same mineral salt medium with and without vitamins (Oraby et al., 2020). Here, both biomass and CL concentrations were only slightly lower when cultivated without vitamins. This also applies to other yeasts, including *Ustilago maydis*, which do not need vitamins for their growth (Burkholder et al., 1944; Kurtzman et al., 2011). Shaking flask experiments with *Sporisorium scitamineum* and *Ustilago maydis* underlined these results and showed that the omission of vitamins may decrease the side-product formation of erythritol (Supplementary Figure S1; Supplementary Table S4). These results show that omitting vitamins from the fermentation medium is a promising means to reduce production costs. It has to be noted, though, that a chelating agent, like citric acid, needs to be added to the trace element components used here to prevent the precipitation of iron after heat sterilisation.

#### 3.4.5 Assessment of Overall Cost Optimisation Potential

Simulation of scenarios 1 to 12 show that increasing the CL concentration and yield, decreasing the fermentation duration, the agitation and aeration rate, using a DSP process without solvents, and omitting vitamins from the fermentation medium all have positive effects on the process economy. In scenario 13, variations of all these parameters were combined and defined as the best-case scenario according to the current state of the art in CL fermentation and showed a possible decrease in UPC of more than 77%. In addition to the reduction in UPC, a significant shift in OC distribution was further observed in the best-case scenario results. Especially the share of labour dependent costs and raw materials became more significant, increasing from 17 to 22% and from 2 to 8%, respectively. This highlights that the further developed/optimized the process becomes, the more important high yields and low raw material/substrate consumption get. This also applies to the scaling effect. A larger fermentation scale and production capacity would result in overall lower UPCs, but the share of raw material/substrate would become even more significant.

At this point, when process-specific optimisation approaches have been exhausted, more general optimisation approaches can lead to further cost reductions. Pinch analysis, for instance, can be performed to decrease energy consumption since the fermentation and purification processes contain various heat-consuming systems that could be used for heat recovery. Another possibility for further cost reduction is switching from batch to a continuous process. Here, less labour is required, and equipment control is simplified (Peters and Timmerhaus, 1991). However, this would require further experimental examinations. A further approach for overall cost reduction is the establishment of multiproduct systems, in which all material inputs and outputs are completely used to generate more profit (Kwan et al., 2018). In the case of CL, using the remaining biomass after CL purification as animal feed or the remaining fructose for the production of purified fructose syrup, for instance, may be a beneficial solution for additional profit generation. However, such general optimisation approaches do not target process optimisation as aimed with our approach, but rather overall economic optimisation.

## 4 Conclusion

The obtained results underline the great potential of process simulation and economic evaluation as a driver for experimental design for process optimisation in early development stages. Comparison of different scenarios of a CL fermentation in a 10 m^3^ scale enabled prioritising the research directed at certain optimisation approaches. For CL, a decrease in fermentation duration, high CL concentrations and yields, a decrease in agitation and aeration rate, the choice of purification method, and utilizing a mineral salt medium with only heat sterilisable components were all identified as parameters affecting process economy. Especially the effect of the different media on the process economy could not have been predicted without economic evaluation. Furthermore, using stoichiometric calculations based on our laboratory data to describe CL fermentation with an *Ustilago maydis* strain, we were able to calculate the maximum possible CL yield Y_P/S_ of 0.45 g_CL_·g_glucose_^−1^ that can be achieved at the biomass yield of 0.10 g_Biomass_·g_glucose_^−1^. This result serves as a foundation for considerations on feeding approach designs to increase CL concentrations in the fermenter.

## Data Availability

The original contributions presented in the study are included in the article/Supplementary Material. Further inquiries can be directed to the corresponding author.
