# Supplementary material for: Techno-Economic Analysis as a Driver for Optimisation of Cellobiose Lipid Fermentation and Purification
Source: Front Bioeng Biotechnol. 2022 Jun 17;10:913351. doi: 10.3389/fbioe.2022.913351 (PMC9249125; doi:10.3389/fbioe.2022.913351)
Supplement: Supplementary file 1 [file DataSheet1.docx]

Supplementary Material

**Supplementary Table 1**: Media components for each cultivation step at their final concentration, acid and base for pH adjustment, and ethanol for cellobiose lipid (CL) extraction, including their prices as used for economic evaluation. Prices were obtained in the year 2020 and possible price fluctuations due to the global COVID-19 pandemic were not considered.

|  | | Component | Con­centra­tion | | | | Price [€/kg] | Approx. Consumption per year for a 10 m3 fermenter [kg] | Source of indicated price |
| --- | --- | --- | --- | --- | --- | --- | --- | --- | --- |
| Seed culture | |  | | [g·L^-1^] |  | | |  |  |
|  |  | Potato dextrose (PD) | | 24 | 126 | | | 96 | estimated based on the price for 500 g  <https://www.neolab.de/de/chemikalien/bio-organische-chemikalien/agar-und-weitere-mikrobiologie-produkte/potato-dextrose-broth-gm403-500g> [accessed 27.08.2020] |
| Production Culture | Vitamins |  | | [µg·L^-1^] | |  | |  |  |
|  |  | p-Aminoben­zoic acid | | 200 | 214 | | | 0.03 | estimated based on the price for 500 g  <https://de.vwr.com/store/product/748684/3-aminobenzoesaure>  [accessed 01.09.2020] |
|  |  | Biotin | | 2 | 41400 | | | <<1 | estimated based on the price for 5 g  <https://de.vwr.com/store/product/2351902/d-biotin-99-reagenzqualitat>  [accessed 01.09.2020] |
|  |  | Calcium panthonate | | 400 | 450 | | | 0.05 | estimated based on the price for 100 g  <https://shop.thgeyer-lab.com/erp/catalog/search/search.action?model.query=pantothens%c3%a4ure>  [accessed 01.09.2020] |
|  |  | Folic acid | | 2 | 6280 | | | <<1 | estimated based on the price for 5 g  <https://shop.thgeyer-lab.com/erp/catalog/search/search.action?model.query=fols%c3%a4ure> [accessed 01.09.2020] |
|  |  | Inositol | | 2000 | 350 | | | 0.27 | estimated based on the price for 100 g  <https://shop.thgeyer-lab.com/erp/catalog/search/search.action?model.query=inositol> [accessed 01.09.2020] |
|  |  | Niacin | | 400 | 90 | | | 0.05 | estimated based on the price for 250 g  <https://de.vwr.com/store/product?keyword=nicotins%C3%A4ure>  [accessed 01.09.2020] |
|  |  | Pyridoxine hydrochloride | | 400 | 771 | | | 0.05 | Price estimated based on the price for 100 g  <https://de.vwr.com/store/product/7195846/pyridoxin-hydrochlorid>  [accessed 01.09.2020] |
|  |  | Riboflavin | | 200 | 184 | | | 0.03 | estimated based on the price for 25 g  <https://de.vwr.com/store/product/2995288/riboflavin-vitamin-b2-technical> [accessed 01.09.2020] |
|  |  | Thiamin Hydrochloride | | 400 | 211 | | | 0.05 | estimated based on the price for 100 g  <https://de.vwr.com/store/product/2995338/thiamin-hydrochlorid-technical> [accessed 01.09.2020] |
|  | Com­pounds Supply­ing Trace Elements |  | | [µg·L^-1^] | |  | |  |  |
|  |  | CuSO_4_ | | 40 | 111 | | | 0.01 | estimated based on the price for 250 g  <https://de.vwr.com/store/product/2383477/kupfer-ii-sulfat-wasserfrei-98> [accessed 01.09.2020] |
|  |  | FeCl_3_ | | 200 | 74 | | | 0.03 | estimated based on the price for 500 g  <https://de.vwr.com/store/catalog/product.jsp?catalog_number=8.03945.1000> [accessed 01.09.2020] |
|  |  | H_3_BO_3_ | | 500 | 26 | | | 0.07 | <https://de.vwr.com/store/catalog/product.jsp?catalog_number=20181.294> [accessed 01.09.2020] |
|  |  | KI | | 100 | 184 | | | 0.01 | estimated based on the price for 500 g  <https://de.vwr.com/store/product/778532/kaliumiodid> [accessed 01.09.2020] |
|  |  | MnSO_4_ | | 400 | 76 | | | 0.05 | <https://de.vwr.com/store/product/22629623/mangan-ii-sulfat-monohydrat-ph-eur-usp> [accessed 01.09.2020] |
|  |  | Sodium molybdate | | 200 | 935 | | | 0.03 | estimated based on the price for 100 g  <https://de.vwr.com/store/product/2998875/natriummolybdat-vi-dihydrat-sigma-aldrich>[accessed 01.09.2020] |
|  |  | Zinc sulfate | | 400 | 93 | | | 0.05 | estimated based on the price for 500 g  <https://de.vwr.com/store/product/2348104/zinksulfat-monohydrat-98-gereinigt> [accessed 01.09.2020] |
|  | Mineral Salts |  | | [g·L^-1^] |  | | |  |  |
|  |  | CaCl_2_ | | 0.1 | 25 | | | 13 | estimated based on the price for 5 kg packages  <https://marketplace.chembid.com/en/calciumchlorid-gekoernt-reinst-5-kg.html> [accessed 01.09.2020] |
|  |  | KH_2_PO_4_ | | 1.0 | 30 | | | 133 | estimated based on the price for 5 kg packages  <https://marketplace.chembid.com/en/di-kaliumhydrogenphosphat-wasserfrei-reinst-ep-5-kg.html> [accessed 01.09.2020] |
|  |  | MgSO_4_ | | 0.5 | 0.720 | | | 66 | estimated based on the price for 1000 kg  <https://www.chemiekaufen.de/Magnesiumsulfat-98-Einzelfuttermittel-Feed/10077495002>  [accessed 01.09.2020] |
|  |  | NaCl | | 0.1 | 0.950 | | | 13 | estimated based on the price for 100 kg packages  <https://marketplace.chembid.com/en/natriumchlorid-72566.html> [accessed 01.09.2020] |
|  | Nitrogen Source |  | | [g·L^-1^] |  | | |  |  |
|  |  | Urea | | 0.6 | 0.205 | | | 80 | calculated from the mean between july 2019 and july 2020 <https://www.indexmundi.com/de/rohstoffpreise/?ware=harnstoff&monate=12&wahrung=eur>) [accessed 27.08.2020] |
|  | Carbon Source |  | | [g·L^-1^] |  | | |  |  |
|  |  | Sucrose  containing Glucose  and Fructose | | 100  50  50 | 0.240  0.580 | | |  | estimated based on stock prices in Germany <https://www.finanzen.net/rohstoffe/zuckerpreis> |
|  |  |  |  |  |  |  |  | 6650 | personal communication with a glucose provider in Germany |
| Other |  | Sulfuric acid (96 %) | |  | 0.120 | | | 21 | personal communication with a sulfuric acid provider in Germany |
|  |  | Sodium hydroxide | |  | 1.658 | | | 21215 | price calculated based on the price of 1200 kg (50 %NaOH), devided by 25 (100 % - 1,658 €)<https://www.chemikalienportal.de/laugen/natronlauge/natronlauge-50.html> [accessed 27.08.2020] |
|  |  | Ethanol | |  | 0.368 | | | 104519 | <https://www.finanzen.net/rohstoffe/ethanolpreis> [accessed 28.08.2020] |

**Supplementary Table 2**: Equipment used for the basic scenario of the CL model. Prices were obtained from the SuperPro Designer database, unless indicated otherwise.

| **Unit Name** | **Size (Capacity)** |  |  | **Unit Price (€/Unit)** |
| --- | --- | --- | --- | --- |
| Air Filter 1 | 3,524.25 | L h^-1^ | | 7,000 |
| Air Filter 2 | 117,571.02 | L h^-1^ | | 7,000 |
| Blending Tank (acidic wash) | 7,482.22 | L | | 185,000 |
| Blending Tank (vitamins) | 10.00 | L | | 46,000 |
| Blending Tank (ethanol extraction) | 2,743.84 | L | | 118,000 |
| Blending Tank (MS + TE + Urea) | 100.15 | L | | 68,000 |
| Centrifugal Compressor (gas) | 30.95 | kW | | 66,000 |
| Condenser | 5.94 | m^2^ | | 31,000 |
| Dead-End Filter | 0.15 | m^2^ | | 2,000 |
| Disk-Stack Centrifuge | 7,001.34 | L h^-1^ | | 97,000 |
| Disk-Stack Centrifuge | 6,956.97 | L h^-1^ | | 97,000 |
| Fermenter | 10,122.57 | L | | 305,000 |
| Flat Bottom Tank (ethanol storage) | 2,788.13 | L | | 90,000 |
| Liquids Drum (acid) | 12.61 | L | | 50,000 |
| Rotary Dryer | 6.95 | m^2^ | | 47,000 |
| Seed Fermenter | 311.34 | L | | 106,000 |
| Thin Film Evaporator | 0.14 | m^2^ | | 84,000 |

**Supplementary Table 3**: Exemplary kLa values measured in a 10 L fermenter with a filling volume of 10 L, using the dynamic-gassing-out method according to^[[1]](#footnote-1)^, while gassing with ambient air using an aeration unit and a ceramic sparger.

| Power input P/V [W m^-3^] | aeration rate [vvm] | agitation rate [rpm] | kLa_aeration ring_ [h^-1^] | kLa_ceramic sparger_ [h^-1^] |
| --- | --- | --- | --- | --- |
| 769 | 0.25 | 500 | 42.96 ± 3.28 | 61.56 ± 0.00 |
| 769 | 0.625 | 500 | 57.72 ± 7.37 | 90.27 ±1.60 |
| 96 | 0.38 | 250 | 20.52 ± 1.06 | n/a |
| 0 | 0.4 | 0 | n/a | 21.24 ± 0.29 |

**Supplementary Table 4**: CL, biomass and erythritol concentrations of two *U. maydis* Δemt1 fermentations in a 1 L fermenter, at 30 °C and pH 2.5 on mineral salt medium with or without vitamins.

| Parameter | with vitamins | without vitamins |
| --- | --- | --- |
| c_x_ [g L^-1^] | 17.4 | 18.7 |
| c_CL_ [g L^-1^] | 8.7 | 9.0 |
| c_Ery_ [g L^-1^] | 13.8 | 10.5 |
| CL:Erythritol | 0.63 | 0.86 |





**Supplementary Figure 1**: Course of CL concentration during shaking flask cultivations (1 L) at 30 °C and pH 2.5 of *S. scitamineum* and *U. maydis* Δemt1 on mineral salt medium with (w) or without (w/o) vitamins.

1. W. S. Wise: The Measurement of the Aeration of Culture Media. Journal of general microbiology (1951) 5, p. 167–177 [↑](#footnote-ref-1)
